# Dysregulated Brain Protein Phosphorylation Linked to Increased Human Tau Expression in the hTau Transgenic Mouse Model

**DOI:** 10.3390/ijms23126427

**Published:** 2022-06-08

**Authors:** Isidro Ferrer, Pol Andrés-Benito, Karina Ausín, Paz Cartas-Cejudo, Mercedes Lachén-Montes, José Antonio del Rio, Joaquín Fernández-Irigoyen, Enrique Santamaría

**Affiliations:** 1 Department of Pathology and Experimental Therapeutics, Network Centre of Biomedical Research of Neurodegenerative Diseases (CIBERNED), Institute of Health Carlos III, University of Barcelona, 08907 Barcelona, Spain; pandres@idibell.cat; 2 Bellvitge University Hospital, Bellvitge Biomedical Research Institute (IDIBELL), Calle Feixa Llarga sn, 08907 Barcelona, Spain; 3 Proteomics Platform, Navarrabiomed, Hospital Universitario de Navarra (HUN), Universidad Pública de Navarra (UPNA), IdiSNA, 31192 Pamplona, Spain; karina.ausin.perez@navarra.es (K.A.); joaquin.fernandez.irigoyen@navarra.es (J.F.-I.); 4 Clinical Neuroproteomics Unit, Navarrabiomed, Hospital Universitario de Navarra (HUN), Universidad Pública de Navarra (UPNA), IdiSNA, Irunlarrea Street, 31192 Pamplona, Spain; pazcarce@hotmail.com (P.C.-C.); mercedes.lachen.montes@navarra.es (M.L.-M.); enrique.santamaria.martinez@navarra.es (E.S.); 5 Molecular and Cellular Neurobiotechnology, Institute of Bioengineering of Catalonia (IBEC), Barcelona Institute for Science and Technology, Science Park Barcelona (PCB), 08028 Barcelona, Spain; jadelrio@ibecbarcelona.eu; 6 Department of Cell Biology, Physiology and Immunology, Faculty of Biology, University of Barcelona, Carrer Baldiri Reixac, 08028 Barcelona, Spain

**Keywords:** hTau, phosphorylation, tau, membrane, cytoskeleton, synapsis, tauopathy

## Abstract

Altered protein phosphorylation is a major pathologic modification in tauopathies and Alzheimer’s disease (AD) linked to abnormal tau fibrillar deposits in neurofibrillary tangles (NFTs) and pre-tangles and β-amyloid deposits in AD. hTau transgenic mice, which express 3R and less 4R human tau with no mutations in a murine knock-out background, show increased tau deposition in neurons but not NFTs and pre-tangles at the age of nine months. Label-free (phospho)proteomics and SWATH-MS identified 2065 proteins in hTau and wild-type (WT) mice. Only six proteins showed increased levels in hTau; no proteins were down-regulated. Increased tau phosphorylation in hTau was detected at Ser199, Ser202, Ser214, Ser396, Ser400, Thr403, Ser404, Ser413, Ser416, Ser422, Ser491, and Ser494, in addition to Thr181, Thr231, Ser396/Ser404, but not at Ser202/Thr205. In addition, 4578 phosphopeptides (corresponding to 1622 phosphoproteins) were identified in hTau and WT mice; 64 proteins were differentially phosphorylated in hTau. Sixty proteins were grouped into components of membranes, membrane signaling, synapses, vesicles, cytoskeleton, DNA/RNA/protein metabolism, ubiquitin/proteasome system, cholesterol and lipid metabolism, and cell signaling. These results showed that over-expression of human tau without pre-tangle and NFT formation preferentially triggers an imbalance in the phosphorylation profile of specific proteins involved in the cytoskeletal–membrane-signaling axis.

## 1. Introduction

Protein phosphorylation is one of the most common and essential reversible post-translational modifications. A phosphorylated amino acid can bind molecules able to interact with other proteins, and activate or inhibit protein function [1]. Phosphorylation also affects protein conformation, protein–protein interactions, and protein signaling [2]. Protein phosphorylation usually occurs in several phosphorylation sites, thus enabling refined molecular functions [3,4]. The balance between protein phosphorylation and dephosphorylation is modulated by the dynamic combined action of specific kinases and phosphatases [5,6,7]. Conversely, abnormal or dysregulated protein phosphorylation may lead to abnormal cellular function involving a variety of vital molecular pathways [8].

Widespread dysregulated protein phosphorylation, in addition to tau hyper-phosphorylation, via extensive kinase activation occurs in Alzheimer’s disease (AD), thus contributing to abnormal function of cell membranes, cytoskeleton, synapses, neurotransmitter receptors, energy metabolism, RNA processing and splicing, protein synthesis, and cell signaling [9,10,11,12,13]. Aberrant protein phosphorylation is already detected at the first stages (stages I–III of Braak) of neurofibrillary tangle (NFT) pathology in regions such as the frontal cortex devoid of NFTs and β-amyloid deposits at these stages [12].

Abnormal protein phosphorylation also occurs in transgenic mouse models of AD that develop β-amyloid deposits in brain [14,15,16,17]. Dysregulated protein phosphorylation in these mice occurs at first stages of the disease and particularly affects synaptic proteins [18,19]. These models are very useful to learn about the core of altered phosphoproteins linked to β-amyloid pathology, but they do not permit the identification of altered proteins due to abnormal tau in AD.

Primary tauopathies are characterized by abundant 3R, 4R or 3R + 4R tau filamentous tau inclusions in neurons and glial cells not associated with extracellular amyloids [20,21]. Some of them are linked to mutations in *MAPT*, the tau gene, but the majority are sporadic. Widespread abnormal protein phosphorylation has been described in a few human tauopathies including aging-related tau astrogliopathy (ARTAG), a common old-age 4Rtauopathy involving astrocytes [22], and globular glial tauopathy (GGT) a rare 4Rtauopathy with characteristic inclusions in neurons, astrocytes, and oligodendrocytes [23].

Several animal models are generated to recapitulate hyper-phosphorylation of tau and the formation of NFTs as key aspects of tauopathies [24,25,26]. P301S transgenic mice express the T34 isoform of microtubule-associated protein tau bearing the human P301S mutation [27]. A recent phosphoproteomics study has been carried out on these mice [28]. The majority of dysregulated phosphoproteins, most of them hyper-phosphorylated, were proteins of the cytoskeleton, membranes, synapses, membrane trafficking, vesicles linked to endo- and exocytosis, cytoplasmic vesicles, and kinases. In contrast, proteins linked to DNA, RNA metabolism, RNA splicing, and protein synthesis were hypo-phosphorylated. Other pathways modulating energy metabolism, cell signaling, Golgi apparatus, carbohydrates, and lipids were also targets of abnormal protein phosphorylation in P301S mice [28].

In addition to tauopathies, a recent classification of diseases with accumulation of tau protein includes a miscellaneous group of various conditions with tau immunoreactivity or tau pathology, but without filamentous tau inclusions [29]. Tau immunoreactivities or tau pathologies can represent, in some cases, a preclinical stage and/or an early cytopathological phase of fibril formation [30].

An interesting model of increased human tau expression and accumulation in the absence of *MAPT* mutations, in a knock-out background for murine tau protein, is hTau [31,32]. hTau transgenic mice express high levels of human 3Rtau and low levels of 4Rtau. hTau mice can also be considered as a model of the human tau expression in the mouse lacking murine tau. Whether these mice can be used as a model of “tau pathology” in humans is open to discussion. However, abnormal deposits in subpopulations of neurons in the cerebral cortex and hippocampus are identified with the antibodies PHF1 and MC1, but tau deposits are not stained with AT8, tau-PSer422, and Tau C-3 antibodies [31,32,33]. Moreover, no abnormal fibrils are observed in mice aged 9 months [31]. Thus, hTau fits with the characteristics of the miscellaneous group in the recent classification of tauopathies and tau pathology/altered tau immunoreactivity [29]. We chose this model to learn about protein modifications, particularly protein phosphorylation linked to tau pathology in the absence of tauopathy.

For this purpose, we used immunohistochemistry and western blotting, label-free (phospho)proteomics, and SWATH-MS to identify differential protein phosphorylation in hTau compared with wild-type mice.

## 2. Results

### 2.1. Immunohistochemical Characterization of hTau Mice

**Figure 1:**
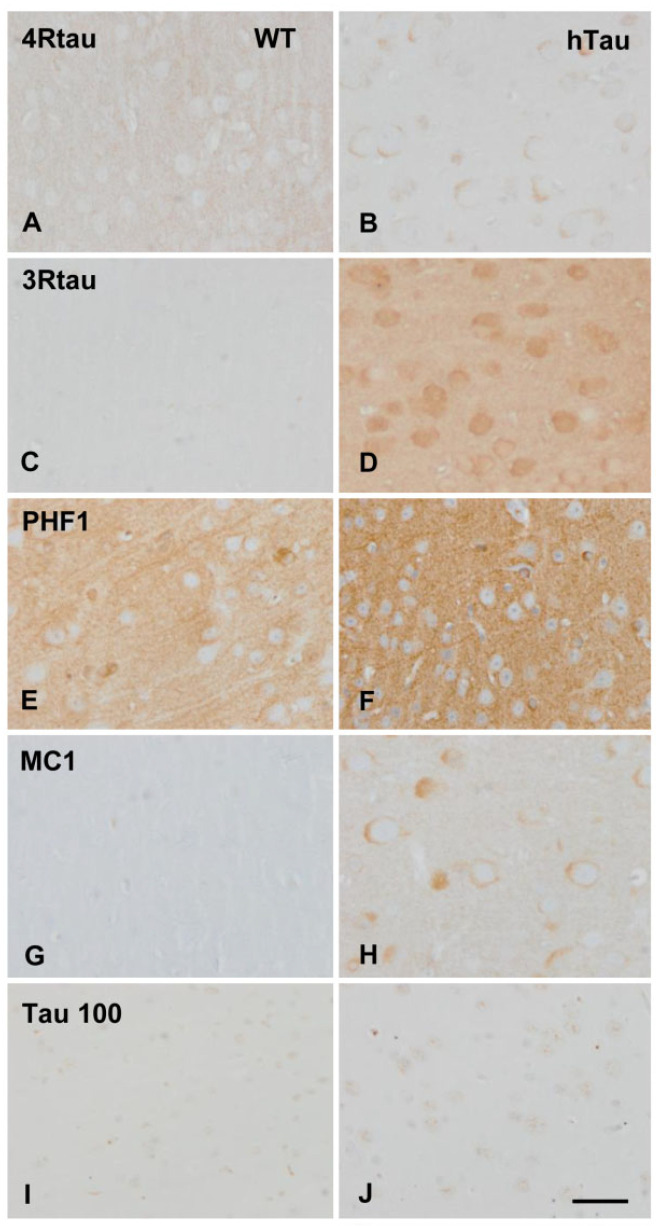
Immunohistochemistry of the cerebral cortex in wild-type (WT) and hTau transgenic mice aged nine months. Weak 4Rtau immunoreacitvity decorates the neuropil in WT (**A**) but weak cytoplasmic neuronal deposits in hTau (**B**). 3Rtau immunostaining is almost absent in WT (**C**) but 3Rtau immunoreactivity is stronger in the neuropil and neuronal cytoplasm in hTau (**D**). PHF1 immunoreactivity is moderate, and MC1 staining is negative in WT (**E**,**G** respectively). In contrast, PHF1 immunostaining is stronger in hTau (**F**), and MC1 antibodies stain the cytoplasm of neurons in hTau (**H**). The antibody Tau 100 weakly stains the nuclei of neurons in WT (**I**), whereas more marked nuclear Tau 100 immunostaining is seen in hTau (**J**). Paraffin sections, lightly counterstained with hematoxylin, bar = 40 µm.

The cerebral cortex of WT mice showed weak 4Rtau immunoreactivity in the neuropil (Figure 1A), and negative 3Rtau staining (Figure 1C); the neuropil and a few neurons showed weak positive PHF1 immunostaining (Figure 1E), but they were negative with MC1 antibodies (Figure 1G). The antibody Tau 100 weakly stained the nuclei of neurons (Figure 1I). In contrast, the cerebral cortex of hTau mice showed weak 4Rtau immunoreactivy in subpopulations of neurons, and moderate 3Rtau immunostaining in the totality of cortical neurons (Figure 1B,D). PHF1 antibodies showed moderate and diffuse immunoreactivity mainly in the neuropil (Figure 1F), and the antibody MC1 immunostained the cytoplasm of a subpopulation of neurons (Figure 1H). The Tau 100 antibody showed more marked nuclear staining in hTau than in WT mice (Figure 1J). No cytoplasmic deposits were detected with the AT8 antibody; truncated tau C-3 immmunostaining was negative. Cytoplasmic deposits were homogeneous without the appearance of pre-tangles or neurofibrillary tangles. Threads were absent. Ubiquitin deposits were not found. Finally, immunostaining with the antibody tau-N Tyr29 was negative in WT and hTau mice. No differences were seen regarding GFAP-positive astrocytes and Iba1-immunoreactive microglia between WT and hTau.

Gallyas staining was negative.

### 2.2. Tau Protein Expression Profile in hTau Brains

**Figure 2:**
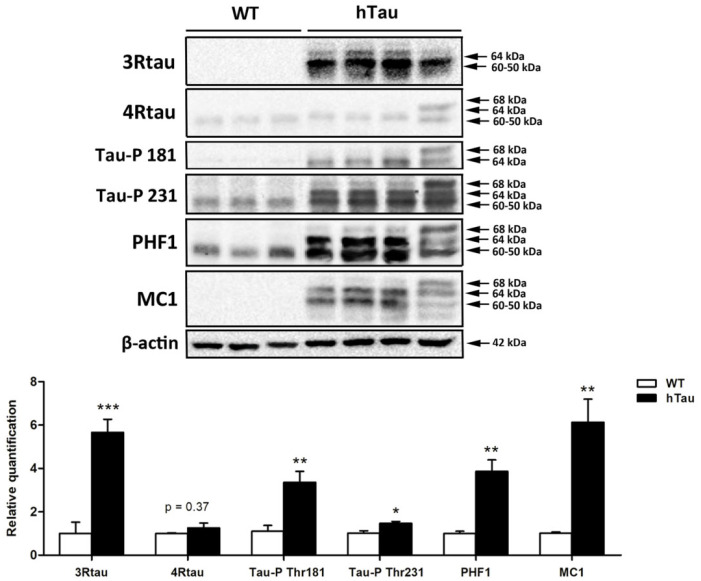
Gel electrophoresis and western blotting of total brain homogenates of 3WT and 4hTau mice processed in parallel. In WT mice, 3Rtau is negative, but two bands of about 64 kDa and 60–50 kDa and a weak band of slightly higher molecular weight is identified in hTau. A weak 4Rtau-immunoreactive band of about 60–50 kDa is identified in WT, and two bands of 68 kDa and 64 kDa in hTau mice. The phospho-tau antibody tau-P Thr181 (Tau-P 181) is negative in WT, but two bands of 68 kDa and 64 kDa are detected in hTau. The phospho-tau antibody tau-P Thr231 (Tau-P 231) recognizes a band of 60–50 kDa and a smear of higher molecular weight in WT, but three strong bands of 68 kDa, 64 kDa, and 60–50 kDa in hTau. PHF1 antibodies detect a unique band of about 64kDa in WT, and three bands of 68 kDa, 64 kDa, and 60–50 kDa in hTau. Finally, MC1 antibodies reveal no bands in WT mice, and several bands of 68 kDa, 64 kDa, and 60–50 kDa in hTau. The slightly different band pattern in hTau mice on the right reflects a higher amount of 4Rtau than in the other hTau mice. Densitometric analysis of western blots show significant differences between WT and hTau regarding the expression levels of 3Rtau, Tau-P Thr181, Tau-P Thr231, PHF1, and MC1. Unpaired student’s *t*-test; significance level set at * *p* < 0.05 ** *p* < 0.01, *** *p* < 0.001. 4Rtau are not significantly increased in hTau (*p* = 0.37) (modified from ref. [33]).

Gel electrophoresis and western blotting of total brain homogenates of 3WT and 4hTau mice were assessed in parallel. In WT mice, 3Rtau immunoreactivity was negative. Two bands of about 64 kDa and 60–50 kDa and a weak band of slightly higher molecular weight were recognized in hTau mice. WT mice showed a weak 4Rtau-immunoreactive band of about 60–50 kDa. Two bands of 68 kDa and 64 kDa were identified in hTau mice. The phospho-tau antibody tau-P Thr181 was negative in WT, but two bands of 68 kDa and 64 kDa were detected in hTau transgenic mice. The phospho-tau antibody tau-P Thr231 recognized a band of 60–50 kDa and a smear of higher molecular weight in WT, and three strong bands of 68 kDa, 64 kDa, and 60–50 kDa in hTau mice. PHF1 antibodies exposed a unique band of about 64 kDa in WT mice but three bands of 68 kDa, 64 kDa, and 60–50 kDa in hTau. Finally, MC1 antibodies revealed no bands in WT mice, and several bands of 68 kDa, 64 kDa, and 60–50 kDa in hTau (Figure 2). Densitometric assessment further revealed significant differences in tau levels between WT and hTau mice (Figure 2).

### 2.3. hTau Expression Induces Phosphoproteomic Changes at Membrane and Synaptic Levels

A total of 2065 proteins were detected in hTau and WT mice (Supplementary Table S1). In addition to protein tau, only five proteins showed increased levels in hTau compared with WT; no proteins were down-regulated in hTau mice. Four proteins were associated with the cytoskeleton: tubulin beta 2B class IIb (Tubb2b), an structural protein of the cytoskeleton and main component of the tubulin complex; transgelin 2 (Tagln2), localized in the cytoskeleton, vesicles, and endosomes which functions as a cadherin-binding protein; glyoxalase domain-containing 4 (Glod4), a cadherin-binding protein localized in mitochondria and extracellular exosomes; and NCK interacting protein with SH3 domain (Nckipsd), a cytoskeletal binding protein implicated in the regulation of actin polymerization and cell adhesion. The fifth up-regulated protein was acetyl-Coenzyme A acyltransferase 1A (Acaa1a), a protein localized in peroxisomes that participates in fatty acid metabolic pathways and unsaturated fatty acid biosynthesis.

Mass-spectrometry revealed increased tau phosphorylation at Ser199, Ser202, Ser214, Ser396, Ser400, Thr403, Ser404, Ser413, Ser416, Ser422, Ser491, and Ser494 in hTau compared with WT mice (Supplementary Table S1).

In addition, 4578 phosphopeptides (corresponding to 1622 phosphoproteins) were identified in hTau and WT mice (Supplementary Table S1). Seventy phosphosites were identified corresponding to 64 differentially phosphorylated proteins in hTau. Sixty proteins were grouped into components of membranes, membrane signaling, synapses, vesicles, cytoskeleton, DNA/RNA/protein metabolism, ubiquitin/proteasome system, cholesterol and lipid metabolism, and cell signaling. The remaining proteins (*n* = 4) cannot be included in one of these groups or their function in the nervous system was not known.

The largest set of differentially phosphorylated proteins (*n* = 37) were linked to membranes, membrane signaling, synapses, vesicles, and cytoskeleton (Table 1). Twenty-four were hyper-phosphorylated and twelve hypo-phosphorylated.

Seven proteins were linked to DNA, RNA, or protein synthesis; four were hyper-phosphorylated, and three hypo-phosphorylated. Six proteins were associated with the UPS; three were hyper- and three hypo-phosphorylated. Four proteins were involved in cholesterol transport and lipid metabolism; two were hypo- and two hyper-phosphorylated. Six proteins had roles in different aspects of cell signaling, five of them hyper-phosphorylated and one hypo-phosphorylated (Table 2).

### 2.4. Interactomes

**Figure 3:**
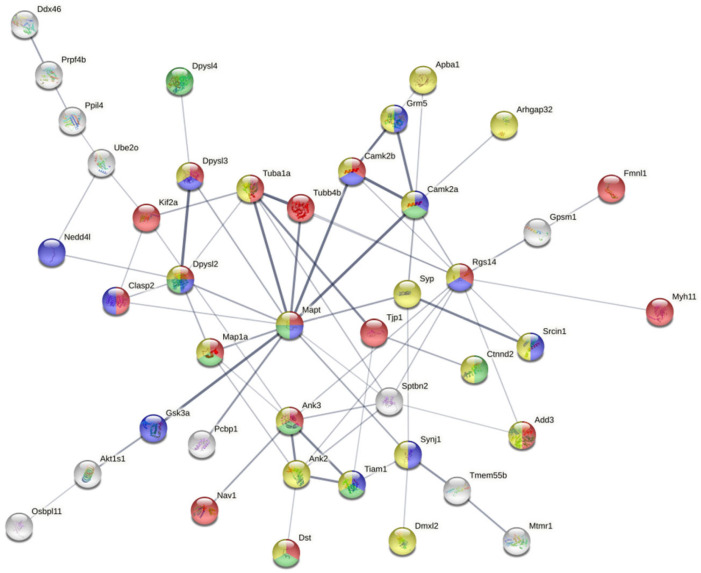
Functional interactome constituted by differential phosphoproteins detected in hTau transgenic compared with WT mice aged nine months; 42 out of 64 differential phosphoproteins (65%) constitute a highly-related functional interactome where most of nodes are involved in cytoskeleton organization, synapse, neuron projection and neurogenesis. Moreover, 12 nodes are considered functional interactors of Tau according to STRING database.

A functional interactome was constructed based on the differential phosphoprotein dataset including tau protein as external input. As shown in Figure 3, 42 out of 64 differential phosphoproteins (65%) constituted a highly related functional interactome where most of nodes were involved in cytoskeleton organization (FDR: 0.00032), synapse (FDR: 6.55 × 10^−5^), neuron projection (FDR: 0.0337), and neurogenesis (FDR: 0.00089). Interestingly, 12 nodes (Tubb4b, Camk2a, Syp, Sptbn2, Synj1, Pcbp1, Gsk3a, Map1a, Clasp2, Dpysl2, Dpysl3, Tuba1a) are considered functional interactors of Tau according to the STRING database.

## 3. Discussion

Increased levels of 3Rtau together with higher tau phosphorylation are the main differences between WT and hTau transgenic mice. Western blotting revealed tau hyperphosphorylation at residues Thr181, Thr231, Ser396/Ser404, and mass spectrometry at residues Ser199, Ser202, Ser214, Ser396, Ser400, Thr403, Ser404, Ser413, Ser416, Ser422, Ser491, and Ser494. However, tau was not phosphorylated at Ser202/Thr205 evinced by the lack of staining with the AT8 antibody; this is not a false negative result as tau inclusions in human and mouse tauopathies show strong AT8 immunoreactivity. Tau in hTau mice had an abnormal conformation, as shown with the antibody MC1 in immunohistochemistry and western blotting. L-ack of immunostaining with the tau C-3 antibody revealed no tau truncation at aspartic acid 421. Gallyas staining was negative. Tau truncation at Asp421 is an early change preceding neurofibrillary tangle formation [34]. Tau ubiquitinitation, which is absent in hTau, is a late tau post-translational event in NFT formation [35]. hTau mice did not contain tau nitrated at Tyr29 which is in contrast with the presence of Tau-N Tyr29 in NFTs, dystrophic neurites, and neuropil threads in AD, and NFTs in corticobasal degeneration and progressive supranuclear palsy [36], and P301S transgenic mice [28].

Tau alterations in hTau here observed were similar to those reported in animals aged 9 months in the original descriptions of hTau although less pronounced with PHF1 and MC1 antibodies [31]. Paired helical filaments, as illustrated in hTau mice aged 13 months [31] were not assessed in our study. Considering morphological and molecular characteristics, hTau transgenic mice largely differ from AD, and whichever of the described human sporadic and familial tauopathies [20,21,29,37,38,39]. Moreover, transgenic mice carrying human tau mutations, as the P301S construct, also differ from hTau mice [27,28,40,41]. Therefore, the present model at the age of nine months is suitable for the study of altered protein expression and dysregulated protein phosphorylation in murine tau knockout mice expressing human 3Rtau and less 4Rtau, and accompanied by tau phosphorylation at selected sites, in the absence of NFTs and pre-tangles. A limitation of the current scenario lies on the lack of control over the modifications due to the knockout of murine tau on which hTau is generated. Curiously, we identified increased levels of only five proteins, in addition to tau, four of them being cytoskeletal proteins, and one a peroxisomal protein that participates in fatty acid metabolism and unsaturated fatty acid biosynthesis.

Regarding the study of phosphorylated proteins, we used a similar equipment and methodological approach as the employed to analyze (phospho)proteomics in AD [12], and primary tauopathies ARTAG [22], GGT [23], and P301S transgenic mice [28].

We identified 230 dysregulated phosphoproteins in the entorhinal cortex and 82 in the frontal cortex at different stages of AD [12].

In ARTAG, 109 proteins were hyper-phosphorylated and 31 hypo-phosphorylated compared with controls. In addition to GFAP that was phosphorylated at positions 8, 14, 82, and 424, many neuronal and glial proteins were differentially phosphorylated in ARTAG. These included proteins of the cytoskeleton, kinases, proteins linked to calcium/calmodulin signalling, cAMP signaling and DNA repair, nuclear and nucleolar regulators, proteins linked to tight junctions, proteins linked to proteolysis, and synaptic proteins. [22]. The study revealed that altered protein phosphorylation in ARTAG was not restricted to astrocytes.

In GGT, phosphoproteome profiling revealed 74 dysregulated proteins in the frontal cortex and 15 in the white matter. Cytoskeleton, axon guidance, exocytosis, chaperone-mediated protein folding, and myelination were part of the significantly over-represented dysregulated biological processes in the frontal cortex. Interestingly, dysregulated specific protein clusters were related to microtubule polymerization, and pre- and post-synaptic transmission [23].

In a parallel study, we identified 328 phosphoproteins differentially regulated in the telencephalon of P301S transgenic mice at the age of 9 months when compared with age-matched WT mice; 179 hyper-phosphorylated, 134 hypo-phosphorylated, and 16 both hyper- and hypo-phosphorylated at different phosphorylation sites [28]. These numbers are quite different from those found in hTau when compared with WT mice in the present study. Seventy proteins were differentially phosphorylated in hTau. Of 60 proteins with known functions in the nervous system, proteins showed an increment in specific phosphosites whereas 19 proteins evidenced a reduction in phosphoprotein levels. Although less marked than in AD, and tauopathies, altered tau expression in hTau mice alters phosphorylation of determined proteins linked mainly with the cytoskeleton, membranes, membrane signaling, synapses, and vesicles; kinases; DNA/RNA/protein synthesis, UPS; and cell signaling.

These targets match with the recently validated principal tau interacting partners (*n* = 153), among total 245 reported tau interacting partners [42], which include proteins involved in phosphorylation and dephosphorylation (*n* = 65), cytoskeleton and intracellular transport (*n* = 24), ubiquitin system and chaperones (*n* = 27), signal transduction (*n* = 11), cleavage and truncation (*n* = 11), and DNA/RNA/protein synthesis (*n* = 4), among others [42].

In addition, we identified four dysregulated phosphoproteins involved in cholesterol transport and lipid metabolism in hTau. This observation is relevant because there is a close functional relationship between cytoskeletal proteins and cell membranes through protein–protein interactions, electrostatic interactions with lipid membranes, and lipid tails. Lipid rafts are microdomains of cell membranes which favor multiple cell signaling interactions at the cell membrane [43]. The exoplasmic leaflet is enriched with glycosphingolipids and sphingomyelin, the cytoplasmic leaflet is enriched with glycerolipids; cholesterol is present in both. The limited number of dysregulated phosphoproteins in hTau transgenic mice aged 9 months is in contrast with the large number of identified proteins forming the phosphorylated tau interactome in the human AD brain [44]. Of 542 proteins identified in microdissected NFTs, 75 interacted with PHF-1-immunoreactive phosphorylated tau, some of them previously associated with total tau [45] but not yet linked to phosphorylated tau [44]. Although we did not perform co-immunoprecipitation studies, it can be assumed that differences between AD and hTau are largely dependent on the limited recruitment of abnormal proteins in hTau neuronal deposits when compared with the abundance of abnormal phosphorylated and non-phosphorylated proteins in NFTs in AD. Moreover, we cannot rule out the possibility that dysregulated phosphoproteins are not trapped by tau deposits in our model. Finally, other abnormally phosphorylated proteins do not apparently interact with tau in AD, human tauopathies, and murine tau models [12,22,23,28].

## 4. Material and Methods

### 4.1. Animals

The experiments were carried out on 9-month-old C57BL/6 WT mice and heterozygous mice expressing human isoforms of tau protein (hTau; B6.Cg- (GFP)Klt Tg(*MAPT*)8cPdav/J) in a C57BL/6 background (Jackson laboratories; reference: RRID:IMSR_JAX:005491; Bar Harbor, ME, USA) [31,32]. This mouse model can only be hemizygous to the transgene as homozygous animals are not viable. The number of mice was eight per group with equal numbers of males and females per group. Transgenic mice were identified by genotyping genomic DNA isolated from tail clips using the polymerase chain reaction conditions indicated by Jackson laboratories. All animal procedures were carried out following the guidelines of the European Communities Council Directive 2010/63/EU and with the approval of the local ethical committee (C.E.E.A: Comitè Ètic d’Experimentació Animal; University of Barcelona, Spain; reference number: 426/18). Animals were killed by cervical dislocation and their brains were then rapidly removed and processed for study. The left cerebral hemisphere was dissected on ice, immediately frozen, and stored at −80 °C until use for biochemical studies. The rest of the brain was fixed in 4% paraformaldehyde, cut in coronal sections, and embedded in paraffin. De-waxed sections were stained with hematoxylin and eosin, Gallyas staining, or processed for immunohistochemistry.

### 4.2. Immunohistochemistry

De-waxed sections, 4 microns thick, were processed for immunohistochemistry as detailed elsewhere [23]. The sections were incubated at 4 °C overnight with one of the primary antibodies listed in Table 3. Control of immunostaining included omission of the primary antibody; no signal was obtained following incubation with only the secondary antibody.

### 4.3. Gel Electrophoresis and Western Blotting

Frozen samples of the posterior part of the left hemisphere from 3 WT and 4 hTau mice were homogenized in RIPA lysis buffer and phosphatase inhibitor cocktail (Roche Molecular Systems, San Jose, CA, USA). The homogenates were centrifuged for 20 min at 12,000 rpm. Equal amounts of protein (12 μg) for each sample were loaded and separated by electrophoresis on 10% sodium dodecyl sulfate polyacrylamide gel electrophoresis (SDS-PAGE) gels and transferred onto nitrocellulose membranes (Amersham, Freiburg, Germany). Incubation in 3% albumin in PBS was used to block non-specific bindings. After washing, membranes were incubated overnight at 4 °C with antibodies detailed in Table 3. β-actin (1:30,000, Sigma, Barcelona, Spain) was used as a control of protein loading. Membranes were incubated with HRP-conjugated secondary antibodies (1:3000, Dako, Barcelona, Spain); the immunoreaction was visualized with a chemiluminescence reagent (ECL, Amersham, UK). Results were analyzed with SPSS 19.0 (SPSS Inc., USA; https://www.ibm.com) software and GraphPad PRISM (GraphPad Software; https://www.graphpad.com) software. Data were presented as mean ± standard error of the mean (SEM). The unpaired student’s *t*-test was used to compare groups. Significance level was set at * *p* < 0.05, ** *p* < 0.01, *** *p* < 0.001.

### 4.4. Phosphoproteomics

Anterior left hemisphere from WT and hTau mice were subjected to phosphoproteome analysis as previously described [12]. The (phospho)proteomes and proteomes were independently analyzed by conventional label-free phosphoproteomics [46] and SWATH-MS (sequential window acquisition of all theoretical fragment ion spectra mass spectrometry) [47], respectively. The High-Select™ TiO_2_ Phosphopeptide Enrichment Kit (Thermo Scientific, Barcelona, Spain) was used to obtain the phosphorylated peptide fractions, according to the manufacturer’s instructions. Protein extraction, in-solution digestion, peptide purification, and reconstitution prior to mass spectrometric analysis were performed as previously reported [12]. Library generation, nanochromatography, mass-spectrometry settings and database searching parameters were as previously described [12]. MS/MS spectra associated to phosphoproteomic data were processed using MaxQuant (1.6.7.0). Database searching was performed using the Uniprot reference for *Mus musculus* (ID: UP000000_10090, 29 July 2021). FDR filter less than 1% was applied at protein, peptide, and PTM level. In the case of SWATH proteome data, MS/MS data acquisition was performed using AnalystTF 1.7 (Sciex, Vaughan, ON, Canada), and spectra files were processed through ProteinPilot v5.0 search engine (Sciex) using the Paragon^TM^ Algorithm (v.4.0.0.0) (https://www.paragon-sofware.com) [48]. A non-lineal fitting method [49] was used to determine the FDR, reporting results with a 1% global FDR or better. Quantitative data were analyzed using the Perseus software (https://maxquant.net) (1.6.14.0) for statistical analysis and data visualization. MS data and search result files are found in Supplementary Table S1.

### 4.5. Bioinformatics

Metascape was used to characterize the significantly dysregulated metabolic pathways based on proteomic profiles [50]. Network analysis was performed using STRING (Search Tool for the Retrieval of Interacting Genes) software (http://stringdb.org/, accessed on 1 April 2022) [51]. All edges were supported by at least one reference from the literature or from canonical information stored in the STRING database. False positives as well as false negatives were minimized using only interactions tagged as “medium confidence” (>0.4) in STRING.

## 5. Conclusions

To sum up, this study showed that over-expression of human tau without pre-tangle and NFT formation in hTau transgenic mice preferentially triggers an imbalance in the phosphorylation profile of specific protein sets involved in the cytoskeletal–membrane-signaling axis rather than a generalized proteome variation.

## Figures and Tables

**Table 1:** Dysregulated phosphorylation of proteins involved in membranes, membrane signaling, synapses, vesicles, and cytoskeleton in hTau compared with WT mice. ↑: up-regulation; ↓: down-regulation.

Symbol	Protein Name	Function	Phosphosite Dysregulation
Add3	Adducin 3	Membrane–cytoskeleton-associated protein that promotes the assembly of the spectrin-actin network	↑
Ank2	Ankyrin-2	Links the integral membrane proteins to the underlying spectrin-actin cytoskeleton	↓
Ank3	Ankyrin-3	Links the integral membrane proteins to the underlying spectrin-actin cytoskeleton	↑
Apba1	Amyloid Beta Precursor Protein Binding Family A Member 1	Vesicular trafficking protein; inhibits production of proteolytic APP fragments	↓
Arhgap23	Rho GTPase Activating Protein 23	Signal transduction through transmembrane receptors	↓
Arhgap32	Rho GTPase Activating Protein 32	NMDA receptor activity-dependent actin reorganization in dendritic spines	↓
ASB3	Ankyrin Repeat And SOCS Box Containing 3	Membrane signaling and cytokine supression	↑
CAMK2A	Calcium/Calmodulin-Dependent Protein Kinase (CaM Kinase) II Alpha	Member of the NMDAR signaling complex in excitatory synapses	↓
CAMK2B	Calcium/Calmodulin Dependent Protein Kinase II Beta	Reorganization of the actin cytoskeleton required for correct targeting of CaMK2A; synaptic plasticity	↓
Cdc42ep1	CDC42 Effector Protein 1	Organization of the actin cytoskeleton	↑
Clasp2	Cytoplasmic Linker Associated Protein 2	Regulation of microtubule dynamics	↓
Ctnnd2	Catenin Delta 2	Adhesive junction associated protein	↑
Dmxl2	Dmx like 2	Scaffold protein on synaptic vesicles.	↑
Dpysl2	Dihydropyrimidinase Like 2	Microtubule assembly, Sema3A-mediated growth cone collapse, synaptic signaling	↑
Dpysl3	Dihydropyrimidinase Like 3	Signaling by class 3 semaphorins, remodeling of the cytoskeleton	↑
Dpysl4	Dihydropyrimidinase Like 4	Signaling by class 3 semaphorins, remodeling of the cytoskeleton	↑
Dst	Dystonin	Anchoring neural intermediate filaments to the actin cytoskeleton	↑
Fmnl1	Formin Like 1	Regulation of cytoskeletal organization	↑
Gpsm1	G Protein Signaling Modulator 1	Signaling from G protein-coupled receptors	↑
GRM5	Glutamate Receptor, Metabotropic 5	Metabatropic glutamate receptor	↓
Kctd16	Potassium Channel Tetramerization Domain-Containing 16	Auxiliary subunit of GABA-B receptors	↑
Kif2a	Kinesin Family Member 2A	Regulates microtubule dynamics during axonal growth	↓
MAP1A	Microtubule-Associated Protein 1A	Microtubule assembly	↓
Myh11	Myosin Heavy Chain 11	Actin binding	↓
Nav1	Neuron Navigator	Axon guidance	↓
Phldb1	Pleckstrin Homology Like Domain Family B Member 1	Microtubule cytoskeleton organization	↑
Rgs14	Regulator Of G Protein Signaling 14	Regulator of G-protein signaling	↑
Slc7a2	Solute Carrier Family 7 Member 2	Membrane protein responsible for the cellular uptake of arginine, lysine and ornithine	↑
SPTBN2	Spectrin Beta Chain, Non-Erythrocytic 2	Component of the cell membrane-cytoskeleton stabilizing the glutamate transporter EAAT4 at the surface of the plasma membrane	↓
SRCIN1	SRC Kinase Signaling Inhibitor 1	Calcium-dependent exocytosis; neurotransmitter release and synapse maintenance	↑
Synj1	Synaptojanin 1	Regulates levels of membrane phosphatidylinositol-4,5-bisphosphate	↑
Syp	Synaptophysin	Membrane protein of synaptic vesicles	↑
Tjp1	Tight Junction Protein 1	Tight junction adaptor protein	↑
Tom1l2	Target Of Myb1 Like 2 Membrane Trafficking Protein	Vesicular trafficking	↑
Tpd52	Tumor Protein D52	Vesicle-mediated transport and clathrin derived vesicle budding	↑
Tuba1a	Tubulin Alpha 1a	Microtubule constituent	↑
Tubb4b	Tubulin Beta 4B Class IVb	Constituent of microtubules	↑

**Table 2:** Dysregulated phosphorylation of proteins linked to DNA, RNA, and protein metabolism; ubiquitin/proteasome system, cholesterol and lipid metabolism, and cell signaling in hTau compared with WT mice.

Symbol	Protein Name	Function	Phosphosite Dysregulation
**DNA/RNA/protein metabolism**			
CARHSP1	Calcium-Regulated Heat-Stable Protein 1	Binds single-stranded DNA	↑
Dars1	Aspartyl-TRNA Synthetase 1	Mediates the attachment of amino acids to their cognate tRNAs	↑
Ddx46	DEAD-Box Helicase 46	Modulates RNA secondary structure	↓
Pcbp1	Poly(RC)-Binding Protein 1	Component of the major cellular poly(rC)-binding protein	↑
PRPF4B	Pre-MRNA Processing Factor 4B	Pre-mRNA splicing and in signal transduction	↓
Ppil4	Peptidylprolyl Isomerase Like 4	Protein folding	↑
Raly	RALY Heterogeneous Nuclear Ribonucleoprotein	Pre-mRNA splicing	↓
**Ubiquitin–proteasome system (UPS)**			
Fbxo41	F-Box Protein 41	Phosphorylation-dependent ubiquitination	↓
Hectd4	HECT Domain E3 Ubiquitin Protein Ligase 4	Component of the E3 ubiquitin-protein ligase	↓
Mindy2	MINDY Lysine 48 Deubiquitinase 2	Hydrolase that can remove ‘Lys-48′-linked conjugated ubiquitin from proteins	↑
Nedd4l	NEDD4 Like E3 Ubiquitin Protein Ligase	Component HECT domain E3 ubiquitin ligases	↑
Psmd9	Proteasome 26S Subunit, Non-ATPase 9	Component of the 26S proteasome	↑
Ube2o	Ubiquitin Conjugating Enzyme E2 O	E2/E3 hybrid ubiquitin-protein ligase that mediates monoubiquitination of target proteins	↓
**Cholesterol and lipid metabolism**			
Gramd1b	GRAM Domain-Containing 1B	Mediates non-vesicular transport of cholesterol from the plasma membrane to the endoplasmic reticulum	↓
Osbpl11	Oxysterol Binding Protein Like 11	Lipid metabolism	↑
Pip4p1/Tmem55b	Phosphatidylinositol-4,5-bisphosphate 4-phosphatase 1	Regulation of cellular cholesterol metabolism; lipid rafts, lysosomal membranes	↑
Relch	RAB11 Binding And LisH Domain, Coiled-Coil And HEAT Repeat Containing	Regulation of intracellular cholesterol distribution from recycling endosomes to the trans-Golgi network	↑
**Cell signaling**			
Akt1s1	Proline-rich AKT1 substrate 1 (Proline-rich AKT substrate)	Subunit of mTORC1	↑
Gsk3a	Glycogen Synthase Kinase 3 Alpha	Multifunctional Ser/Thr protein kinase	↑
MTMR1	Myotubularin-Related Protein 1	Contains the consensus sequence for the active site of protein tyrosine phosphatases	↑
Ppp1r14a	Protein Phosphatase 1 Regulatory Inhibitor Subunit 14A	Phosphatase, higher inhibitory activity when phosphorylated	↑
RTN4	Reticulon 4	Endoplasmic reticulum, involved in neuroendocrine secretion or in membrane trafficking	↑
Tiam1	TIAM Rac1-Associated GEF 1	RAC1-specific guanine nucleotide exchange factor	↓

**Table 3:** Antibodies used for immunohistochemistry and western blotting.

Antibody	Supplier	Reference	Host	WB Dil	IHQ Dil
3Rtau	Upstate	05-803	Ms	1/1000	1/800
4Rtau	Millipore	05-804	Ms	1/1000	1/50
tau-P Thr181	Thermo Scientific	PA1-14413	Rb	1/1000	-
tau-P Thr231	Calbiochem	577813	Rb	1/1000	-
PHF1, tau-P Ser396/Ser404	Dr. Peter Davies	-	Ms	1/250	1/500
tau AT8-P Ser202/Thr205	Innogenetics	90206	Ms	-	1/50
tau MC1	Dr. Peter Davies	-	Ms	1/250	1/20
tau C-3	Millipore	36-017	Ms	-	1/100
Tau-N Tyr29	Millipore	MAB2244	Ms	-	1/200
Iba1	Wako	019-19741	Rb	-	1/1000
GFAP	Dako	Z0334	Rb	-	1/400
β-actin	Sigma	A5316	Ms	1/30,000	-
ubiquitin	Dako	Z0458	Ms	-	1/250

Upstate—Syracuse, NY, USA; Millipore, Merck—Burlington, MA, USA; Thermo Scientific—Waltham, MA, USA; Calbiochem—San Diego, CA, USA; Innogenetics—Gent, Belgium; Wako—Richmond, VA, USA; Dako—Santa Clara, CA, USA; Sigma—Burlington, MA, USA.

## Data Availability

Not applicable.

## Supplementary Materials

The following supporting information can be downloaded at: https://www.mdpi.com/article/10.3390/ijms23126427/s1.
